# Effect of Dietary Paeoniae Radix Alba Extract on the Growth Performance, Nutrient Digestibility and Metabolism, Serum Biochemistry, and Small Intestine Histomorphology of Raccoon Dog During the Growing Period

**DOI:** 10.3389/fvets.2022.839450

**Published:** 2022-04-04

**Authors:** Jing Wang, Guangyu Li, Wei Zhong, Haihua Zhang, Qianlong Yang, Lihong Chen, Jinming Wang, Xuewen Yang

**Affiliations:** ^1^Institute of Special Animal and Plant Sciences, Chinese Academy of Agricultural Sciences, Changchun, China; ^2^College of Animal Science and Technology, Qingdao Agriculture University, Qingdao, China; ^3^College of Animal Science and Technology, Hebei Normal University of Science and Technology, Qinhuangdao, China; ^4^China Animal Husbandry Group, Beijing, China

**Keywords:** paeoniae radix alba extract, production performance, nutrients digestibility and metabolism, serum biochemical parameters, raccoon dog, small intestinal histomorphology

## Abstract

Paeoniae radix alba extract (PRA extract) has the functions of regulating immunity, resisting inflammation, and has antioxidant properties. However, current recommendations of dietary PRA extract levels for raccoon dogs were inadequate. The purpose of this experimental study was to gain information allowing for better estimating the effects of PRA extract on raccoon dogs, and their PRA requirements. Fifty healthy male raccoon dogs of (120 ± 5) days old were randomly divided into 5 groups (group PRA0, PRA1, PRA2, PRA4, PRA8) with 10 animals in each group and 1 in each replicate. Five kinds of experimental diets were prepared with five levels of Paeoniae radix alba extract (0, 1, 2, 4, 8 g/kg) in the basic diet. The prefeeding period was 7 days and the experimental period was 40 days. The results showed that the average daily feed intake in group PRA1 and PRA2 was significantly higher than that in other groups (*P* < 0.01). The dry matter excretion in group PRA8 was significantly higher than that in other groups (*P* < 0.01), while the dry matter digestibility and protein digestibility in group PRA8 were significantly lower than those in other groups (*P* < 0.01). Nitrogen retention in group PRA1 and PRA2 was significantly higher than that in group PRA8 (*P* < 0.05). With the increase of the content of Paeoniae radix alba extract in diet, the activity of alkaline phosphatase in group PRA2 was significantly higher than that in group PRA0 (*P* < 0.05); The activity of serum SOD in group PRA4 was significantly higher than that in other groups (*P* < 0.01). The content of serum IgA in group PRA2 was significantly higher than that in other groups (*P* < 0.05). The content of TNF-α in intestinal mucosa in group PRA1 and group PRA2 was significantly lower than that in group PRA0 (*P* < 0.05). In conclusion, we found that dietary Paeoniae radix alba extract intake significantly improved the feed intake and nitrogen deposition of Ussuri raccoon dog, increased the content of serum IgA and reduced the content of TNF-α in the small intestinal mucosa. We suggest that an estimated dietary Paeoniae radix alba extract level of 1 to 2 g/kg could be used as a guide to achieve the optimal performance of raccoon dogs.

## Introduction

Ussuri raccoon dog (*Nyctereutes procyonoides*) originated in China and is an economically valuable fur animal. The breeding capacity of raccoon dogs in China can reach 40 million, which generates tremendous economic benefits for the country. Animal intestinal mucosa has a barrier function, which can prevent invasion of pathogenic antigens (1). If the intestinal mucosa of a raccoon dog is damaged, the absorption of nutrients by animals will be affected, and the resistance of the intestinal tract to pathogenic bacteria will be reduced (2). Thus, the health of animals will be influenced and the production performance greatly decline. In production practice, intestinal diseases affect the health of raccoon dogs and often cause huge economic losses. The small intestine is located in the middle of the digestive tract, which can be exposed to a large number of bacteria and pathogens from both the upper and lower digestive tracts due to abnormal intestinal movement (3), making the animal is very susceptible to illness. Inhibiting inflammation and oxidative stress were helpful for alleviating intestinal diseases in animals (4).

Many studies have shown that in animal feeding, compound Chinese herbal medicine has the functions of improving animal growth performance, nutrient digestibility, antioxidant capacity and immune capacity, and so on. Paeoniae radix alba (PRA) is often a component of compound Chinese herbal medicine. PRA is the root of Paeonia lactiflora pall which has shown rich medicinal value for more than 2000 years (5–7). Previous studies have shown that the PRA extract can improve immunity, improve antioxidant capacity, and alleviate inflammatory bowel disease in rats and mice (8, 9). There have been numerous clinical reports on the efficacy and safety of PRA extract in various diseases. The adverse events of PRA extract were mainly gastrointestinal tract disturbances, mostly mild diarrhea. Most of the events were self-resolved in 1–2 weeks. No adverse events on hepatic, renal, or hematological tests were found (10). According to Announcement No. 1773 feed raw material catalog of the Ministry of Agriculture of the People's Republic of China, PRA belongs to a natural plant that can be fed to animals. Ussuri raccoon dog is an omnivorous animal, and its diet generally contains a certain proportion of plant raw materials, which shows that PRA can be used as the feed component of raccoon dogs. However, current recommendations of dietary PRA extract levels for raccoon dogs were inadequate. Therefore, the purpose of this study was to explore the effect of PRA extract alone in the raccoon dog diet and the optimal addition level. So, we investigated the effects of dietary PRA extract levels on the growth performance, nutrient digestibility and metabolism, serum biochemistry, and small intestine histomorphology of Ussuri raccoon dog during the growing period.

## Materials and Methods

### Experimental Animals

The key field scientific observation and test station of Changbai Mountain Wildlife Resources, owned by the Ministry of Agriculture, was chosen as the feeding trial site. Fifty healthy male raccoon dogs of about 120 ± 5 days old were randomly selected and divided into five groups (group PRA0, PRA1, PRA2, PRA4, PRA8). Racoon dog litters were split up between groups to eliminate the influence of genetic factors. There were 10 replicates in each group and one in each replicate. There was no significant difference in weight among the groups (*P* > 0.05).

### Experimental Design and Experimental Diet

All animals were weighed prior to the experiment and randomly distributed to five experimental groups employing the completely randomized design (CRD), and subjected to diets with PRA extract levels of 0 g/kg (control group, PRA0), 1 g/kg (PRA1), 2 g/kg (PRA2), 4 g/kg (PRA4), and 8 g/kg (PRA8). According to the optimal concentration of PRA extract to alleviate various diseases in rats in previous studies, and the body weight and the daily feed intake of raccoon dog in this study, the conversion factor of mg/kg to mg/m^2^, the appropriate addition level converted into diet might be 1.0–3.2 g/kg. Therefore, the doses of 1, 2, and 4 g/kg were selected. In order to observe whether the excessive dose has a negative impact on raccoon dogs, a dose of 8 g/kg was also selected. The PRA extract was purchased from Nanjing Dasf biotechnology co., Ltd. The PRA extract was determined by HPLC, according to the peak area ratio of sample and standard substance, the content of paeoniflorin in PRA extract was 11.86%, and the content of albiflorin was 3.92%. Referring to NRC (1982) (11) and related literature (12, 13) about the nutrient requirements of raccoon dogs during the breeding period, the experimental diets were prepared with extruded corn, soybean cypress, extruded soybean, rice bran cypress, DDGS, fish meal, meat and bone meal, chicken meal, hemoglobin powder, glucose, and soybean oil as raw materials. The composition and nutrition level of the diets were shown in Table 1. The pre-feeding period lasted for 7 days and the trial lasted for 40 days.

**Table 1:** Composition and nutrient levels of diets (air-dry basis) %.

**Items**	**Content**
**Ingredients**	
Fish meal	1.5
Meat and bone meal	4
Chicken meal	2
Hemoglobin powder	1.5
Extruded corn	40.7
Soybean meal	15
Extruded soybean	5
Rice bran meal	12
DDGS	11
Soybean oil	3.5
Glucose	1
CaHPO_4_	0.6
NaCl	0.5
Premix^a^	1
Methionine	0.2
Lysine	0.5
Total	100
**Nutrient levels (%)**	
Metabolizable energy/(MJ/kg)^b^	14.03
Crude Protein	23.99
Ether Extract	6.56
Ca	0.94
Total P	0.89

a *The premix provided the following for per kg diet: VA 6,500 IU; VD_3_ 1,300 IU; VE 70 IU; VK_3_ 2 mg; VC 103 mg; VB_1_ 12.5 mg; VB_2_ 9 mg; VB_6_ 7.5 mg; VB_12_ 0.02 mg; Biotin 0.1 mg; Folic acid 0.5 mg; nicotinic acid 25 mg; Calcium pantothenate 17.5 mg; Cu 18 mg; Fe 96 mg; Zn 78 mg; Mn 48 mg; I 1.44 mg; Se 0.3 mg; Cr 0.24 mg*.

b *The ME was a calculated value while the others were measured values*.

### Feeding and Management

Before the study, the raccoon dogs were immunized routinely. In the study, raccoon dogs were raised in a single cage. They were fed by specially assigned personnel once at 07:30 and 14:30 every day. They had free access to food and water. The breeding houses were kept clean and dry. The health status of raccoon dogs was observed and recorded every day.

### Digestion and Metabolism Trail

The digestion and metabolism trails were carried out from September 19, 2020, to September 21, 2020, for 3 days. Using the total fecal collection method, the feces collected in 3 days were mixed evenly, sprayed with a small amount of 10% sulfuric acid solution, dried at 65°C to constant weight, ground through a 40-mesh sieve, and preserved. Before urine collection, 10% sulfuric acid 20 mL was added to the collection bucket to fix nitrogen. The 3-day urine was evenly mixed and filtered and stored at −20°C.

### Preparation of Serum and Small Intestine Samples

On the 36th day after the beginning of the feeding experiment, each group of raccoon dogs had blood taken in the morning (before feeding). Blood samples were collected with a 5 ml procoagulant tube. The samples were quickly transferred to the laboratory, and centrifuged for 10 min by 3,500 r/min. The separated serum was packed in a 1.5 mL Eppendorf tube and stored at −80°C. Raccoon dogs were euthanized and the small intestinal tissue was collected and stored in liquid nitrogen immediately, then transferred to −80°C for further testing.

### Determination Index and Method

After the beginning of the trial period, weight on the first day was recorded as the initial weight, and weight at the end of the experiment was recorded as the final weight, the average daily gain (ADG) of each raccoon dog was calculated. The daily given feed and leftover feed of each raccoon dog was recorded so that the average daily feed intake (ADFI) and feed-gain weight ratio (Feed/Gain, F/G) were calculated. The content of dry matter, crude protein, crude fat, and crude ash in basic diet was measured, along with feces (14). Additionally, the protein content in urine was measured.

The calculation method was as follows:


$$\begin{array}{lll} \text{Average}\;\text{daily}\;\text{gain}\;\text{(ADG)} & = & =\frac{\text{final}\;\text{weight}-\text{initial}\;\text{weight}}{\text{test}\;\text{days}}\\ \text{Average}\;\text{daily}\;\text{feed}\;\text{intake}\;\text{(ADFI)} & = & \frac{\text{sum}\;\text{of}\;\text{daily}\;\text{feed}\;\text{intake}}{\text{test}\;\text{days}}\\ \text{Feed}-\text{gain}\;\text{weight}\;\text{ratio}\;\text{(F/G)} & = & \frac{\text{average}\;\text{daily}\;\text{feed}\;\text{intake}}{\text{average}\;\text{daily}\;\text{gain}}\\ \text{Nutrients}\;\text{digestibility}\;\left(\%\right) & = & \frac{\text{nutrient}\;\text{intake}-\text{total}\;\text{fecal}\;\text{nutrients}}{\text{nutrient}\;\text{intake}}\times 100\\ \text{Nitrogen}\;\text{deposition}\;\text{(g/d)} & = & \text{intake}\;\text{nitrogen}-\text{fecal}\;\text{nitrogen}-\text{urinary}\;\text{nitrogen}\\ \text{Net}\;\text{protein}\;\text{utilization}\;\text{rate}\;\left(\text{NPU}\right)\left(\%\right) & = & \frac{\text{nitrogen}\;\text{deposition}}{\;\text{intake}\;\text{of}\;\text{nitrogen}}\times 100\\ \text{Protein}\;\text{biological}\;\text{value}\;\left(\text{PBV}\right)\left(\%\right) & = & \frac{\text{nitrogen}\;\text{deposition}}{\text{nitrogen}\;\text{intake}-\text{fecal}\;\text{nitrogen}}\times 100 \end{array}$$

Serum lactate dehydrogenase (LDH), Aspartate aminotransferase (AST), Alanine aminotransferase (ALT), and Alkaline phosphatase (ALP) were determined by automatic biochemical analyzer Selectra E (the Netherlands). Serum superoxide dismutase (SOD) activity, glutathione peroxidase (GSH-Px) activity, malondialdehyde (MDA) content, IgA, IgG, and IgM were determined by Nanjing Jiancheng kit. Serum D-Lactate (D-lac), Diamine oxidase (DAO), jejunal mucosal interleukin-18 (IL-18), and Tumor necrosis factorα (TNF-α) were detected by double-antibody sandwich enzyme-linked immunosorbent assay (microplate reader, BioTek, Vermont, USA).

### Histological Examinations

The small intestine tissue soaked in neutral formaldehyde was washed with running water for more than 2 h and dehydrated with gradient alcohol. The fixed tissue was embedded in paraffin and sectioned into 5-μm thickness. For hematoxylin and ecosin (H&E) staining, the slices were dewaxed and stained with hematoxylin (Solaibao, China) for 5 min. and stained with eosin (Sangon Biotech, China) for 3 min. The staining was observed under an optic microscope (Olympus, Japan).

### Data Processing and Analysis

The data were processed by Excel 2016, and a one-way analysis of variance (one-way ANOVA) was carried out by SAS 9.4 software GLM program. The results were expressed as “mean ± standard deviation.” Duncan's method was used for multiple comparisons. *P* < 0.01 means extremely significant difference, *P* < 0.05 means significant difference, and *P* > 0.05 means that the difference was not significant.

## Results

### Growth Performance

The effects of dietary PRA extract levels on the growth performance of raccoon dogs were shown in Table 2. There were no significant differences in final weight, ADG, and F/G among treatments (*P* > 0.05), indicating a minimal effect of PRA extract on the growth of raccoon dogs. In contrast, PRA extract level had a significant effect on ADFI (*P* < 0.01). Raccoon dogs in the PRA1 groups and PRA2 groups had higher ADFI than those in other groups (*P* < 0.01).

**Table 2:** Effects of dietary PRA extract levels on growth performance of raccoon dog.

**Items**	**Initial weight/kg**	**Final weight/kg**	**Average daily gain/(g/d)**	**Average daily feed intake /(g/d)**	**Feed/Gain**
PRA0	4.85 ± 0.34	7.09 ± 0.49	56.13 ± 6.47	286.43 ± 1.55^Bc^	5.16 ± 0.58
PRA1	4.82 ± 0.26	7.01 ± 0.41	54.75 ± 7.14	288.78 ± 2.77^Ab^	5.36 ± 0.70
PRA2	4.82 ± 0.37	7.08 ± 0.39	56.63 ± 4.29	290.63 ± 1.10^Aa^	5.16 ± 0.39
PRA4	4.76 ± 0.33	7.00 ± 0.47	55.88 ± 5.95	285.94 ± 1.99^Bc^	5.17 ± 0.59
PRA8	4.75 ± 0.41	6.86 ± 0.48	52.75 ± 4.89	284.12 ± 1.51^Bd^	5.43 ± 0.47
*P*-value	0.9631	0.7793	0.5963	<0.0001	0.7294

*In the same column and the same item, values with no letter or the same letter superscripts mean no significant difference (P > 0.05), while with different small letter superscripts mean significant difference (P < 0.05), and with different capital letter superscripts mean significant difference (P <0.01). The same as below in Tables 3–8*.

### Nutrient Digestibility

The effects of PRA extract supplementation on nutrient digestibility are shown in Table 3. Dietary PRA extract supplementation decreased the digestibility of DM, and CP (*P* < 0.01). Raccoon dogs in the PRA8 groups had higher dry matter excretion than those in t other groups (*P* < 0.01). Besides, the dry matter digestibility and protein digestibility of group PRA8 were significantly lower than those of other groups (*P* < 0.01). The level of dietary PRA extract had no significant effect on the fat digestibility of raccoon dogs (*P* > 0.05).

**Table 3:** Effects of dietary PRA extract levels on nutrients digestibility of raccoon dog.

**Items**	**DM output/g**	**DM digestibility/%**	**Protein digestibility/%**	**Fat digestibility/%**
PRA0	79.64 ± 6.93^Bb^	72.19 ± 2.48^Aa^	68.94 ± 3.10^Aa^	83.88 ± 7.60
PRA1	78.31 ± 7.65^Bb^	72.88 ± 2.66^Aa^	70.22 ± 2.75^Aa^	82.80 ± 4.60
PRA2	82.76 ± 8.71^Bb^	71.52 ± 3.06^Aa^	68.03 ± 3.98^Aa^	87.13 ± 3.88
PRA4	83.30 ± 7.14^Bb^	70.86 ± 2.60^Aa^	67.64 ± 3.05^Aa^	84.75 ± 4.46
PRA8	92.80 ± 7.84^Aa^	67.33 ± 2.85^Bb^	63.06 ± 1.98^Bb^	82.52 ± 7.63
*P*-value	0.0011	0.0004	<0.0001	0.4166

### Nitrogen Metabolism

As shown in Table 4, there was a very significant difference among the nitrogen intake groups of raccoon dogs (*P* < 0.01). It was significantly higher in group PRA2 than in group PRA0 and PRA1, and nitrogen intake in groups PRA0 and PRA1 was significantly higher than that in groups PRA4 and PRA8 (*P* < 0.01). There was a very significant difference among fecal nitrogen groups (*P* < 0.01) and group PRA8 was significantly higher than that of other groups (*P* < 0.01). There was a significant difference among nitrogen deposition groups (*P* < 0.05), that in group PRA1 and PRA2 was significantly higher than that in group PRA8 (*P* < 0.05). There was no significant difference in urinary nitrogen, NPU, and PBV between the five groups (*P* > 0.05).

**Table 4:** Effects of dietary PRA extract levels on nitrogen metabolism of raccoon dog.

**Items**	**Nitrogen intake/(g/d)**	**Fecal nitrogen/(g/d)**	**Urine nitrogen/(g/d)**	**Nitrogen retention/(g/d)**	**Net protein utilization rate/%**	**Protein biological value/%**
PRA0	11.06 ± 0.06^Bb^	3.43 ± 0.33^Bb^	4.28 ± 0.51	3.34 ± 0.48^ab^	30.20 ± 4.22	43.83 ± 5.90
PRA1	11.12 ± 0.11^Bb^	3.31 ± 0.30^Bb^	3.91 ± 0.63	3.89 ± 0.75^a^	35.01 ± 6.80	49.74 ± 8.79
PRA2	11.23 ± 0.04^Aa^	3.59 ± 0.44^Bb^	3.72 ± 0.84	3.92 ± 0.85^a^	34.91 ± 7.58	51.31 ± 10.99
PRA4	10.88 ± 0.08^Cc^	3.52 ± 0.33^Bb^	3.98 ± 0.72	3.38 ± 0.63^ab^	31.03 ± 5.84	45.97 ± 9.04
PRA8	10.84 ± 0.06^Cc^	4.00 ± 0.20^Aa^	3.79 ± 0.68	3.05 ± 0.60^b^	28.13 ± 5.54	44.68 ± 8.94
*P*-value	<0.0001	0.0004	0.4054	0.0223	0.0592	0.5670

### Serum Biochemical Indexes

#### Serum Enzyme Indexes

The effects of different dietary PRA extract levels on serum enzyme indexes of raccoon dogs were shown in Table 5. There was no significant difference among LDH, AST, and ALT. There was a significant difference in ALP between groups. With the gradual increase of the content of PRA extract in diet, the activity of ALP increased at first and then decreased, and the activity of ALP in group PRA2 was significantly higher than that in group PRA0 (*P* < 0.05).

**Table 5:** Effects of dietary PRA extract levels on serum enzyme index of raccoon dog.

**Items**	**LDH/(U/L)**	**AST/(U/L)**	**ALT/(U/L)**	**ALP/(U/L)**
PRA0	82.32 ± 18.20	25.68 ± 4.59	45.26 ± 12.85	36.76 ± 9.86^b^
PRA1	77.74 ± 13.99	28.80 ± 7.76	42.34 ± 23.62	41.87 ± 7.19^ab^
PRA2	69.22 ± 22.39	33.38 ± 13.27	39.22 ± 15.55	50.39 ± 7.56^a^
PRA4	61.20 ± 15.62	32.55 ± 9.79	26.17 ± 8.13	42.08 ± 8.40^ab^
PRA8	77.27 ± 26.87	35.89 ± 10.19	45.84 ± 26.60	44.87 ± 8.74^ab^
*P*-value	0.2537	0.2488	0.2267	0.0404

#### Serum Antioxidant Indexes

As shown in Table 6, different dietary PRA extract levels had a very significant effect on the serum SOD activity of raccoon dogs, and group PRA4 was significantly higher than other groups (*P* < 0.01). However, the serum GSH-PX activity and MDA content of raccoon dogs were not affected by the level of dietary PRA extract (*P* > 0.05).

**Table 6:** Effects of dietary PRA extract levels on serum antioxidant indexes of raccoon dog.

**Items**	**SOD activity(U/mL)**	**GSH-PX (U/L)**	**MDA content (nmol/ml)**
PRA0	43.23 ± 6.50^Bb^	1787.36 ± 108.65	4.83 ± 0.99
PRA1	41.96 ± 8.51^Bb^	1775.62 ± 131.81	4.60 ± 0.52
PRA2	43.90 ± 7.42^Bb^	1903.46 ± 121.90	4.40 ± 0.77
PRA4	76.26 ± 16.58^Aa^	1859.54 ± 153.31	4.79 ± 1.09
PRA8	51.51 ± 7.73^Bb^	1827.19 ± 220.58	4.53 ± 0.58
*P*-value	<0.0001	0.4465	0.8215

#### Serum Immune Indexes

As shown in Table 7, different dietary PRA extract levels had no significant effect on serum IgG and IgM of the raccoon dogs (*P* > 0.05) but there were significant effects on serum IgA (*P* < 0.05). The level of IgA in group PRA2 was significantly higher than that in other groups (*P* < 0.05).

**Table 7:** Effects of dietary PRA extract levels on serum immune indexes of raccoon dog.

**Items**	**IgA ug/ml**	**IgG mg/ml**	**IgM ug/ml**
PRA0	127.02 ± 9.15^b^	1.06 ± 0.10	61.83 ± 2.84
PRA1	124.99 ± 9.20^b^	1.05 ± 0.12	67.42 ± 4.14
PRA2	142.68 ± 18.79^a^	1.01 ± 0.11	67.97 ± 7.86
PRA4	127.67 ± 10.48^b^	0.95 ± 0.09	66.27 ± 5.53
PRA8	128.49 ± 8.68^b^	1.03 ± 0.10	66.31 ± 8.15
*P*-value	0.0376	0.2967	0.2988

### Small Intestinal Permeability and Inflammatory Factors

As shown in Table 8, different dietary PRA extract levels had no significant effect on the activities of D-Lac and DAO in the serum of raccoon dogs (*P* > 0.05). Different dietary levels of PRA had no significant effect on the content of IL-18 in the small intestinal mucosa of the raccoon dogs (*P* > 0.05) but had a significant effect on the content of TNF-α in the small intestinal mucosa of raccoon dog (*P* < 0.05). The content of TNF-α in group PRA1 and group PRA2 was significantly higher than that in group PRA0.

**Table 8:** Effects of dietary PRA extract levels on small intestine of raccoon dog.

**Items**	**D-Lac nmol/ml**	**DAO ng/ml**	**IL-18 ug/L**	**TNF-α ng/ml**
PRA0	13.77 ± 3.38	22.95 ± 4.39	2.25 ± 0.79	8.17 ± 1.68^a^
PRA1	13.25 ± 0.55	15.06 ± 3.89	1.89 ± 0.87	3.41 ± 1.12^b^
PRA2	10.89 ± 2.53	14.65 ± 5.34	1.61 ± 1.06	4.57 ± 2.27^b^
PRA4	13.45 ± 1.68	19.21 ± 3.76	2.11 ± 0.42	5.88 ± 1.94^ab^
PRA8	13.36 ± 4.73	19.81 ± 7.86	2.02 ± 0.20	5.42 ± 1.86^ab^
*P*-value	0.6502	0.1921	0.7852	0.0252

### Small Intestinal Histomorphology

**Figure 1:**
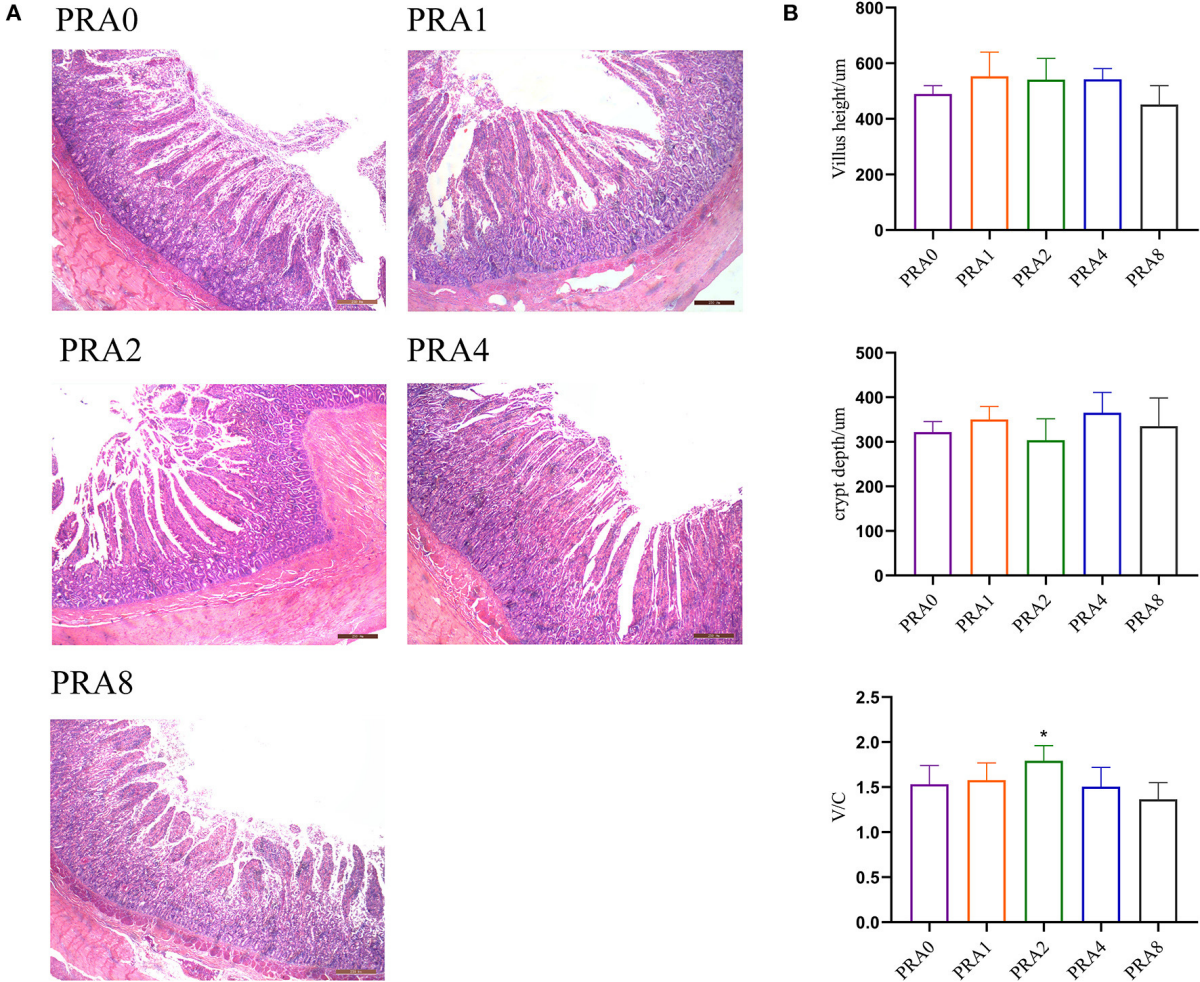
Effects of dietary Paeoniae Radix Alba on intestinal morphology of raccoon dog. **(A)** HE stained sections of the small intestine (40 ×). **(B)** Villus height, crypt depth, and their ratio of small intestine *P < 0.05 vs. group PRA0.

As shown in Figure 1A, there was less enteritis cell infiltration in the small intestine in each group. As shown in Figure 1B, there was no significant difference in villus height and crypt depth among the groups (*P* > 0.05). Compared with the group PRA0, the ratio of villus height to crypt depth in group PRA1and group PRA2 increased significantly (*P* < 0.05).

## Discussion

### Growth Performance

At the end of the experiment, there was no significant difference in the final weight, average daily gain, and feed-to-weight ratio among different groups, which indicated that different dietary PRA extract levels had no effect on the growth performance of raccoon dogs. In recent years, the research of PRA extract was mainly focused on its pharmacological effects, such as analgesia, anti-inflammation, antibacterial, anti-oxidation, anti-cancantiliver fibrosis, anti-autoimmune diseases, anti-cardiovascular diseases, anti-cerebrovascular diseases, and anti-neurodegenerative diseases (5, 7, 9, 15). PRA extract is mostly in the form of compatibility with other traditional Chinese medicine to form a compound when it was added to the feed. The research has shown that the addition of Chinese herbal medicine residue composed of (Radix astragali, Radix angelicae sinensis, Radix rehmanniae preparata, Paeoniae raidix alba, and etc.) could promote the growth and development of piglets to a certain extent, especially fermented Chinese herbal medicine residue (16). Furthermore, the compound prescription of Chinese herbal medicine containing PRA was also studied in sows. Dietary supplementation with the herb residues (from Radix astragali, Radix angelicae sinensis, Radix rehmanniae preparata, Raidix paeoniae alba, and etc.), during the perinatal period improved the reproductive performance in sows (17). CZKJKL (composed of saikosaponins, poly-saccharide of Atractylis macroceohala, TGP) can efficiently relieve weanling stress in piglets (18).

In this experiment, PRA extract was added to the diet, not compound Chinese herbal medicine, which did not play a role in promoting the growth of raccoon dogs. However, with the increasing of the content of PRA extract, the average daily feed intake of the raccoon dogs increased at first and then decreased, indicating that PRA extract had a certain effect on the palatability of diet, but the palatability of diet will decrease when the content of PRA extract was too high.

### Nutrient Digestibility

The nutrient digestibility of raccoon dogs can affect the growth of animals. The main reasons that the addition of Chinese herbal medicine to feed can improve the absorption of nutrients in animals can be listed as follows: 1. Chinese herbal medicine is rich in nutrients, such as amino acids, minerals, vitamins, etc., which can make the diet composition better and more balanced, so as to improve the feed conversion efficiency (19). 2. Chinese herbal medicine has a local stimulating effect, which can promote intestinal peristalsis and improve the digestion and utilization of diet (20). 3. Chinese herbal medicine contains digestive enzymes, such as lipase, amylase, sugar invertase, and proteolytic enzyme, which can effectively promote animal digestion of diet (21). 4. Chinese herbal medicine can adjust the structure of intestinal flora, improve the intestinal environment of animals, promote the reproduction of beneficial bacteria, prevent intestinal villus atrophy, and promote the absorption of nutrients by intestinal mucosa (22–27). In this study, the addition of PRA extract did not promote the digestion of nutrients compared to group PRA0 (0 g/kg PRA extract). The reason may be that the PRA extract plays a role in relieving inflammation and improving immunity, so it failed to promote the absorption of nutrients in the intestine. However, when the supplementary amount was 8 g/kg, the PRA extract did not have a negative effect on the growth of raccoon dogs, which may be due to the animals themselves being in a healthy state.

### Nitrogen Metabolism

The feed intake of animals determines the amount of nitrogen intake. The protein was mainly absorbed in the small intestine. Nitrogen metabolism reflects the utilization rate of protein by raccoon dogs. Net protein utilization and biological value of protein were used to measure the degree of protein utilization in feed and the protein requirement of animals. The research showed that the addition of Chinese herbal medicine (30% pine needles, 20% mugwort, 40% garlic, and 10% Astragalus mongholicus as dry matter) to the diet could decrease fecal and urinary N contents and increased N retention of Mongolian lambs (28). Moderate dietary CTHM (Fructus Ligustri Lucidi seed, Radix Astragali root, Radix Codonopsis root) supplementation (300–500 mg/kg DM) has been reported to increase the non-ammonia N flux in the small intestine and absorption of essential amino acids in growing sheep (21). In this experiment, PRA extract supplemented with 1 and 2 g/kg did not significantly improve the nitrogen deposition of raccoon dogs, which was consistent with the results of their growth performance. This may be because the extract of PRA extract has no effect on the absorption and utilization of amino acids. However, when the amount of PRA extract was added to 8 g/kg, the nitrogen deposition of raccoon dogs decreased, and there was no significant difference in net protein utilization and protein biological value groups, which may be due to a decrease in nitrogen intake and an increase in nitrogen excretion in the high dose group. The average daily gain of raccoon dogs in the 8 g/kg PRA extract group was not significantly lower than that in other groups, however, it was still suggested that it was not suitable to add a high dose of PRA to the diet for a long time.

### Serum Indexes

#### Serum Enzyme Indexes

LDH is an important oxidoreductase in the glycolysis pathway, which exists widely in animals. The abnormal content of LDH in serum can reflect the pathological changes of the myocardium and liver to some extent. AST is mainly distributed in the myocardium. When the cardiomyocytes are damaged, the cell membrane permeability increases, and the content of AST in blood increases. ALT is mainly found in all kinds of cells, especially in hepatocytes. When hepatocytes have pathological changes, ALT is released into the blood in large quantities, so the content of ALT in blood increases. Therefore, the contents of AST and ALT in serum can be used as markers of myocardial and liver injury. PRA extract has a hepatoprotective effect and can effectively protect against acute-on-chronic liver failure (29), liver fibrosis (30), liver cancer (31), and so on. Its mechanism may be the joint action of a variety of signal pathways (7). PRA extract significantly decreased serum ALT and AST activities in Male Sprague-Dawley rats who with non-alcoholic steatohepatitis (NASH) (32). In this experiment, the contents of serum LDH, AST, and ALT in each group were within the normal range, besides, there was no significant difference among all the groups. It indicated that PRA extract had no effect on improving myocardial and liver function when raccoon dogs were in a healthy state. The ALP in the serum of growing animals mainly comes from bones. The intensity of osteogenesis was positively correlated with the activity of ALP. In this experiment, the content of ALP in serum increased at first and then decreased which indicated that adding an appropriate amount of PRA extract to the diet was helpful to promote bone calcification and stimulate bone growth.

#### Serum Antioxidant Indexes

Many kinds of enzymes can reflect the anti-reaction ability of an animal body, such as T-SOD, GSH-Px, MDA, and so on (33). When exposed to oxidative stress, PRA protected H9c2 cells against decreased antioxidant capacity by increasing the content of intracellular GSH (34). Moreover, paeoniflorin can protect human EA.hy926 endothelial cells against gamma radiation-induced oxidative injury by activating the NFE2-related factor 2/heme oxygenase-1 pathway (35). In this experiment, adding 4 g/kg PRA extract to the diet can improve the activity of SOD in serum which indicates that adding the appropriate amount of PRA extract to the diet can enhance the antioxidant capacity of the body. This may be related to the presence of flavonoids and triterpenes in PRA extract. Appropriate flavonoids can be used as reductants and hydrogen donors in the redox reaction of animal bodies. They improve the ability of antioxidation by neutralizing oxygen free radicals and scavenging hydrogen peroxide and superoxide ions produced by oxidative stress (36). Appropriate triterpenes have antioxidant activity and can prevent liver alcohol injury by increasing the activities of SOD, CAT, and GPX and reducing the level of MDA (37, 38).

#### Serum Immune Indexes

After being stimulated by antigens, animal bodies can produce immunoglobulins that interact specifically with antigens. The main three kinds of immunoglobulins in serum are IgG, IgM, and IgA, their contents can reflect the immune function of animals to various bacteria and viruses. The antigenic material in the small intestine is absorbed by microfold cells (M cell). After a series of immune reactions, the main immunoglobulin produced is IgA. IgA forms secretory IgA (sIgA), which is endocytosis by absorbing cells and released into the intestinal cavity. SIgA can strongly resist the decomposition of digestive enzymes and specifically bind to antigens, so as to inhibit or kill bacteria, neutralize viruses, and prevent antigens from adhering to and penetrating into the epithelium. PRA extract can exert anti-inflammatory and immunomodulatory effects in many ways, such as G-protein-coupled receptors (GPCRs) signaling pathway, NF-κB signaling pathway, MAPKs signaling, B lymphocytes, T lymphocytes, and dendritic cells, etc., (39). In this experiment, adding appropriate PRA extract to the diet can increase the content of IgA in the serum of raccoon dogs. The conclusion could be drawn that PRA extract could improve the humoral immune function of raccoon dogs and their resistance to external adverse environments.

### Small Intestinal Permeability and Inflammatory Factors

The intestinal barrier function can be indirectly reflected by detecting intestinal permeability (40). The level of D-lac in peripheral blood can reflect the changes in intestinal mucosal damage and permeability (41). The level and activity of DAO in peripheral blood can reflect the injury and repair of the intestinal mucosal epithelium (42). Research has shown that Curcumin, a Chinese herbal medicine, can reduce the levels of D-lactic acid and DAO in the blood of rats with enteritis induced by methotrexate (MTX) (43). Treatment with glycyrrhizic acid significantly reduced D-lac but did not inhibit DAO activity in MTX-induced enteritis (44). In this experiment, with the PRA extract, there was no significant change in the levels of D-lac and DAO in peripheral blood, because the raccoon dogs were in a healthy physiological state and their intestinal mucosa was not damaged. IL-18 is mainly produced by activated macrophages and epithelial cells in the intestine. It plays an important role in the process of immune injury (45–47). TNF-α can participate in the intestinal mucosal inflammatory response and play an important role in promoting the necrosis and exfoliation of intestinal mucosal epithelial cells (48). TNF-α can also increase the permeability of local intestinal mucosa by reducing the TJs protein in the tight junction between cells (49). Paeoniflorin is one of the effective components of PRA extract. Some studies have shown that paeoniflorin inhibited the levels of TNF-α both in sepsis model rats *in vivo* and RAW264.7 cells *in vitro* (50). Furthermore, paeoniflorin inhibited atherosclerotic inflammatory cytokines IL-1β, IL-6, and TNF-α via the blockade of the TLR-4-mediated NF-κB signaling path-way (51). Other results indicate that paeoniflorin protects mice against lethal LPS challenge, at least in part, through inhibiting TNF-α and IL-1β production and accelerating IL-10 expression (52). In this experiment, different dietary levels of PRA extract had no significant effect on the level of IL-18 in the small intestine of the raccoon dog, but the added level of 1–2 g/kg PRA extract had a significant effect on the level of TNF-α, indicating that the PRA extract has a certain regulatory effect on the intestinal immune stress response of raccoon dog.

### Small Intestinal Histomorphology

The villus length, crypt depth, etc. of the small intestine are important indexes to measure the digestion and absorption function of the small intestine. As the length of the villi increases, the number of absorbing cells on the villi increases, and the contact area between the small intestine and nutrients is larger, which is more conducive to nutrient absorption. crypt depth of the Small intestinal primarily reflects the rate of epithelial cell update rate. The ratio of villus length to crypt depth may comprehensively reflect the functional status of the small intestine. The increase of the ratio indicates that the digestion and absorption capacity of the small intestine is enhanced. Plant extracts have a certain effect on improving the intestinal histomorphology of animals. Research (53) has shown that resveratrol increased the ratio of villus height to crypt depth, increased the number of goblet cells, and reduced the histopathological damage of jejunum of ducks caused by acute heat stress. Dietary supplementation with *Ampelopsis grossedentata* extract facilitated nutrient adsorption in hens via intestinal histology changes for the reason that the villus height in duodenum and villus height to crypt depth ratio in duodenum and jejunum of *A. grossedentata* extract group was significantly higher than that of basal diet group (54). Piglets from Forsythia suspensa extract-fed sows had higher villus height in the ileum, and villus height to crypt depth ratio in jejunum and crypt depth in ileum compared with those from sows fed a control diet (55). In this study, from the villus height, crypt depth, and their ratio in the small intestine of Ussuri raccoon dog, there was no significant difference between the diet supplemented with groups PRA extract and the group control, though there was still a trend of enhanced digestion and absorption capacity of the small intestine. This might be related to the different physiological stages and health status of animals. It can only explain the preventive effect of PRA extract to a certain extent, the follow-up observations based on IBD raccoon dog models might be further helpful in clarifying the effects of PRA extracts.

## Conclusion

In conclusion, we found that dietary Paeoniae radix alba extract intake significantly improved the feed intake and nitrogen deposition of Ussuri raccoon dog, increased the content of serum IgA and reduce the content of TNF-α in the small intestinal mucosa. We suggest that an estimated dietary Paeoniae radix alba extract level of 1–2 g/kg could be used as a guide to achieve the optimal performance of raccoon dogs.

## Data Availability Statement

The original contributions presented in the study are included in the article/supplementary materials, further inquiries can be directed to the corresponding author/s.

## Ethics Statement

The animal study was reviewed and approved by Animal Care and Use Guidelines of the Institute of Special Animal and Plant Science.

## Author Contributions

JingW conceived and designed research, managed the animals, analyzed the data, and drafted the manuscript. WZ, HZ, and GL conceived and designed research, reviewed and edited the manuscript. QY, LC, JinmW, and XY collected the sample and conducted the experiments. All authors contributed to the article and approved the submitted version.

## Funding

This research was funded by the Science and Technology Innovation Program of the Chinese Academy of Agricultural Sciences (CAAS-ASTIP-2022-ISAPS), Special Animal Feeding and Comprehensive Utilization Science and Technology Innovation Center of Jilin Province (20190902015TC), Start-up Fund for Scientific Research of High-level Talents of Qingdao Agricultural University (1121009), and Shandong Modern Agricultural Technology & Industry System (SDAIT-21-01).

## Conflict of Interest

XY was employed by the China Animal Husbandry Group. The remaining authors declare that the research was conducted in the absence of any commercial or financial relationships that could be construed as a potential conflict of interest.

## Publisher's Note

All claims expressed in this article are solely those of the authors and do not necessarily represent those of their affiliated organizations, or those of the publisher, the editors and the reviewers. Any product that may be evaluated in this article, or claim that may be made by its manufacturer, is not guaranteed or endorsed by the publisher.
